# Patients’ views about treatment with combination therapy for Rheumatoid Arthritis: a comparative qualitative study

**DOI:** 10.1186/1471-2474-13-200

**Published:** 2012-10-18

**Authors:** Heidi Lempp, Darija Hofmann, Stephani L Hatch, David L Scott

**Affiliations:** 1 Academic Department of Rheumatology, King’s College London School of Medicine, Weston Education Centre, 10, Cutcombe Rd, London, SE5 9RJ, UK; 2 Department of Psychological Medicine, King’s College London, Institute of Psychiatry, Weston Education Centre, 10, Cutcombe Rd, London, SE5 9RJ, UK

**Keywords:** Combination treatment, Qualitative study, Rheumatoid arthritis

## Abstract

**Background:**

Combinations of disease-modifying anti-rheumatic drugs (DMARDs) are increasingly used to control active rheumatoid arthritis (RA); however there is little information about patients’ perspectives, their expectations, concerns and experiences of this intensive treatment.

**Method:**

We interviewed a quota sample of 18 patients from a single tertiary outpatient clinic, stratified by gender, ethnicity and age, based on the outpatient clinic population. Patients with early RA (<2 years) received combined conventional DMARDs; patients with established RA (>2 years) received combined conventional DMARDs or DMARDs with biologics.

**Results:**

Four main themes emerged from the analytical framework: (i) patients’ expectations about the combined treatment focuses mainly on physical symptoms; (ii) the impact of the treatment on quality of life varied with the new medication in both groups (iii) concerns about new interventions concentrated mainly on potential side effects; and (iv) combination therapy can be self-managed in close collaboration with clinic staff, but this requires individualised management approaches. These themes resonate with von Korff’s collaborative management of chronic illness model.

**Conclusion:**

To our knowledge this is the first qualitative study that examined systematically in patients with early and established RA their expectations, impact on quality of life, concerns about side effects and the management of the treatment when taking combined medication with DMARDs or DMARDs and biologics. Patients have generally positive views of combination DMARDs. Within routine practice settings, achieving medication concordance with complex combined DMARD regimens is challenging, and the concerns vary between patients; careful individual assessments are essential to successfully deliver such intensive treatment.

## Background

Combination therapy has been proven through randomised controlled trials as effective in the early disease process of Rheumatoid Arthritis (RA) [1-3]. It is now accepted that initial combination therapy and biologic agents are more effective than single disease-modifying anti-rheumatic drugs (DMARDs) [4]. However biologics are not the first treatment of choice in early RA, combination of DMARDs is the first option. The debilitating effect of early RA, short-term functional impairment is mostly caused by local and systemic inflammation, whereas long-term untreated inflammation leads to structural, functional and irreversible damage [5]. There have been concerns amongst rheumatologists about the side-effects, risks and complications of DMARDs and the newer biologics [1]. There are major differences in the costs of treatments, with most conventional DMARDs costing less than £1,000 per year, whilst most biologics including tumour necrosis factor (TNF) inhibitors, cost almost £10,000 per year [6]. In addition health expectations of patients also play a role what treatment can be realistically offered [7]. Three recent qualitative studies [1,7,8] tried to capture the views of providers and recipients of this new treatment and its combination. Patients’ responses to the newly offered treatment in early RA tended to be more positive in comparison to rheumatologists who expressed a degree of reluctance in prescribing lots of medication. This divergence is not new in RA care, as highlighted in the literature in relation to physical and psycho-social aspects of rheumatology care and its management [9-12]. This draws attention to how clinician rated treatments may not coincide with the views of patients with RA for example. In return such disagreement may interfere with patients’ cooperation when responding to their individually tailored needs. Against this background of current sea change in the treatment of RA, the aim of our study was to explore expectations, the impact on quality of life, concerns and management of patients with newly diagnosed and established RA when in receipt of combination therapy.

## Method

We decided to undertake a qualitative interview study to obtain greater detailed understanding of patients’ daily experiences, expectations and potential difficulties managing their combination therapy. During outpatient clinic time patients are routinely asked to complete questionnaires, e.g. Health Assessment Questionnaires, Disease Activity and Visual Analogue Scores to assess their clinical outcomes. In discussion with patients they expressed lack of motivation over time in filling in these measures. In their opinion the given answers do not reflect the daily reality of living with RA and self-management. Obtaining of qualitative data via focus groups was also considered, but proofed difficult to arrange due to patients other commitments, e.g. work, family duties, caring for others.

### Recruitment of patients

We studied a quota sample of 18 patients, stratified by gender, ethnicity and disease duration (early RA diagnosis (< 2 years) receiving combinations of DMARDs; or established RA (>2 years) receiving either combinations of DMARDs or TNF inhibitors combined with methotrexate, or an alternative DMARDs, based on the Rheumatology outpatient clinic population (see Table 1). All attended a single tertiary outpatient clinic. The DMARDs used in combination therapy included methotrexate, hydroxychloroquine, sulfasalazine, leflunomide, intramuscular gold and cyclosporine. Steroid injections or oral prednisolone were offered to patients as occasional supplementary treatment; they were not a routine part of combination therapy. The duration of receiving combination therapy ranged from 4 months to 6 years in the study cohort. All patients met the American College of Rheumatology and European League against Rheumatism criteria for RA [13,14].

**Table 1:** Socio-demographic characteristics of study cohort (=18 participants)

	
**Gender**	14 women, 4 men
**Age range, mean and median**	Range: 21–70 years, mean: 49 years, median: 50 years
**Self-described ethnicity**	1 Asian; 1 Black African; 1 Black British;1 British Bengali; 1 Caribbean; 1 Columbian; 11 White British/English; 1 Welsh
**Employment status**	Employed full-time *x*2; employed part-time *x*2; self-employed x1; student fulltime x1; housewife *x*2; retired x5; unemployed x5.
**Registered disabled**	Yes: 12; No: 6
**Range of year of diagnosis (median)**	1966-2011 (2007)
**Early RA (<2 years)/established RA (> 2 years)**	8 patients/10 patients
**Receipt of combined DMARDs/combined DMARDs & biologic treatment**	13 patients/5 patients with RA
**Range of duration of combined treatment (median)**	4 - 96 months (36 months)

Apart from the diagnosis and combined medication, the inclusion criteria were: 18 years of age or older, and able to understand and communicate in English. Exclusion criteria: being seriously ill, or having learning, hearing or communication difficulties. Eight female patients refused to take part, due to lack of time (x5); moving abroad (x1); being on mono-therapy (x1); and being too unwell on combined treatment (x1).

### Interview conduct

Participants were interviewed according to their expressed preference by one researcher (HL) either face to face in a private room in the Medical School (x14) before or after their outpatient clinic appointment; or at home (x1) or by telephone (x3) when their RA was less well controlled that resulted in limited mobility. Prior to the interview the researcher obtained a written informed consent from each interviewee. Each interview took on average of 20–30 minutes.

The interview guide was derived from the literature [8,11] and experiential knowledge of one author with clinical responsibility (see Appendix 1). Three main questions were the focus of the study, followed up by supplementary ones: (i) what led to the decision to commence you on combination therapy; (ii) what is your understanding of the combined treatment; (iii) how do you assess your quality of life since your commenced the combination therapy.

A pilot study was conducted with two patients, after which they were asked to provide feedback about the length of the interview, feasibility, comprehensiveness and relevance of the interview schedule [15]. Both pilot participants positively endorsed those criteria. In addition, one clinician (DS) assessed the content of the two interviews and confirmed that no further amendments to the interview guide were required. Both pilot interviews were therefore included in the main study. Our study protocol specified to approach 10–20 outpatient attendees with RA and when saturation of themes was reached following 18 interviews, further recruitment of participants was discontinued [16,17].

### Data analysis

All interviews were transcribed by two authors (HL, DH) and uploaded to the qualitative computer software programme NViVO 9 [18]. Qualitative content analysis [19] was applied, including single counting and inclusion of negative findings [20,21]. After the initial coding, an external experienced qualitative researcher (SLH) cross-checked the codes and an agreement between both researchers (HL, SLH) was reached. Following the second and third coding phase, the emerging themes were again discussed with the external researcher, the analytical framework identified and the theoretical concept endorsed [22].

This meant that the known topics were grouped into four interlinked headings (i) expectations about combined therapy; (ii) impact of treatment on quality of life; (iii) concerns about combined treatment and (iv) its management at home. Patients’ accounts in relation to their treatment offered and prescribed resonated well with the central concept of von Korff’s collaborative management of chronic illness [23]. Elements of this notion in relation to self-care [24-27], provided an important context and substantiated the relevance and meaning for the key findings of our study [22]. Local Research Ethics Committee and Research & Development approvals were obtained.

## Results

Patients with early RA (8/18) all received combined DMARDs. Participants with established RA (10/18) received either combined DMARDs (5/18) or DMARDs combined with biologics (5/18). Detailed inspection of the qualitative data identified four emerging themes within the analytical framework, which showed some differences between patients with early (n = 8) and established RA (n = 10). These themes comprised: (i) patients’ expectations about combined therapy; (ii) the impact of treatment on quality of life; (iii) concerns about combined treatment; and (iv) management of combined therapy at home.

### Theme 1: Patients’ expectations about combined therapy

All patients (18/18) described in detail how and why the decision was taken to prescribe combined treatment. Combined therapy was commenced either because the disease was not well controlled (10/18), and/or the mono-therapy was not effective enough (8/18). All patients had experienced treatment failure at different stages of their illness trajectory.

"‘… so that is my story, I have been given combination therapy and the doctor suggested this was the path I would follow, this was the new approach now. And quite honestly I was delighted to go along… I was getting quite limited, it was impossible to fill up the kettle or put the tap on’. (Patient 9, early RA)"

"‘My blood always showed inflammation, every drug I had taken did not control the inflammation. I started off with methotrexate [mtx] more than 24 years ago, but I still had pain. One drug just did not work for me. So the medical staff started me on Sulfasalazine and I took it for one month but it did not agree with me. Then they offered me anti-TNF [treatment] and that really suited me and my life style’. (Patient 5, established RA)"

When asked what kind of expectations they had about the combination therapy, regardless whether the interviewees had early or established RA, all (18/18) focused on the desire to improve their physical symptoms, e.g. pain, mobility, fatigue and insomnia, followed by qualifying that these positive enhancements would allow them to maintain some degree of independence to carry out activities of daily living and/or to continue with paid work. 7/18 participants stated that their expectations were not met, which caused dissatisfaction and ongoing physical discomfort (See Table 2).

**Table 2:** Random examples of accounts of met and unmet expectations of 6/18 patients who commenced on combination therapy

**Met expectations (n = 7)**	**Unmet expectations (n = 11)**
‘I thought it could not work for me, but in fact it did, the most scaring thing for me was not being able to walk or not being able to do anything. But now… I can do a lot of things that I could not do before, well, housework, vacuum cleaning, cooking, and a lot of few bits that I am happy to do. (Patient 4, established RA)	‘I am a bit disappointed now, the pain is sort of coming back more strongly now; and I had my jab [steroid injection] today…I find it difficult to walk, and my ankles get weak and in my fingers I don’t have the strength’. (Pilot Patient 1, established RA)
‘My main expectations are: being able to sleep through the night and not being woken at 3am in the morning in pain, ability to work, I have to function and clarity of mind; I suppose that these are the important improvements I expect from the treatment’. (Patient 7, established RA)	‘I was expecting to get better, like giving me more freedom with my health, mobility is the main thing and I think if you are in pain your mood is different and it changes, you feel you are not well yourself’. (Patient 3, early RA)
‘When I started taking the medicines at the beginning, they started working immediately. I thought I would feel better very, very soon and it happened [mobility fatigue], so I think all my expectations were met’. (Patient 12, established RA)	‘The combination therapy has helped, but not as much as I hoped it would. I think I was hoping for a miraculous change [pain, mobility] and it did not happen’. (Patient 8, established RA)

### Theme 2: The impact of treatment on quality of life

Patients’ assessments of whether their treatment was working varied in both early and established subgroups. The impact on their physical symptoms tended to dominate, e.g. pain, swelling, stiffness, mobility and fatigue. In the early RA group, 1/8 patients was not satisfied, 2/8 were unsure and 5/8 expressed satisfaction. In participants with established RA, 1/10 stated the combined treatment did not work (received DMARDs and biologics), 3/10 expressed uncertainty and 6/10 stated contentment (of which four received DMARDs and biologics). Random examples from each group and category are summarised in Table 3.

**Table 3:** Random examples of statements about whether combination therapy is working by 6/18 participants

**Treatment is working**	**Unsure whether treatment is effective**	**Treatment is not working**
***Patient with early RA (n = 1 unsatisfied, n = 2 unsure, n = 5 satisfied)***		
‘I can move easier and some days I do not even remember that I got RA’. (Patient 13)	‘I would have thought the ‘good’ period lasted 5–6 months, apart from the feet. But the hands, you know, swelling up again, but in the initial stages I’ve got to say it [combination therapy] did help, definitely’. (Patient 3)	‘When I take the medication I do not feel any change. I feel tired and I am in bed most of the time. If I want to go out sometimes I cannot go, because I am really, really tired’. (Patient 4)
***Patients with established RA (n = 1 unsatisfied, n = 3 unsure, n = 6 satisfied)***		
‘Stiffness and pain level is much better than it was, and generally the scores [ESR and Disease Activity Score] and the feelings are the same’. (Patient 10)	‘The treatment is working, I am pain free and do not have so much deformity.. I find it easier to pull myself out of the bath, but there again I have been given a steroid injection, and the steroid does help’. (Pilot Patient 2)	‘Stiffness and pain are the same since I started the combined treatment’. (Pilot Patient 1)

In relation to physical pain, a dominant symptom in RA, one female felt no pain at all. The majority used phrases such as ‘pain relief’, ‘ease of pain’; ‘discomfort’,’cannot get rid of the pain’, ‘some pain’; ‘control of disease’; ‘not being pain free’; no pain free future’.

The reports about their quality of life since commencement of combined therapy resulted in mixed descriptions: two patients were undecided, 12/18 provided positive answers (of which four received DMARDs and biologics), and 4/18 were less favourable, of which one patient was on DMARDs and biologics (which coincided with the ones who stated unmet expectations). The broad World Health Organisation (1997) definition of Quality of Life was adopted in the study that related to a person’s physical health, psychological state, level of independence, social relationships, personal beliefs and their relationship to salient features of their environment. Participants made positive and negative references to health issues, e.g. pain, control of RA, mobility, side effects but also to their independence, personal relationships and change in their social life.

"‘hm… no change [my quality of life] since I started to be sick, because once you get RA, your life changes’. (Patient 3, early RA)"

"‘I am undecided [about my quality of life]; I got into a routine with my life’. (Patient 10, established RA)"

"‘The RA is completely out of sight, the medication is now controlling it, I am so much better, and I met a man and I eventually married him, the relationship is so much easier, because we can do many things together’. (Patient 16, established RA)"

### Theme 3: Concerns about combined treatment

When asked about their concerns about the offered and prescribed combined treatment, many (14/18) expressed several worries about the medication and its potential side effects. These ranged from ambivalence (e.g. positive and negative impact), additional physical problems (e.g. deteriorating eye sight, damage to internal organs); anxiety about cancer with methotrexate (e.g. immune suppression, perceived as ‘strong medication); uncertainty about treatment failure (e.g. controlling RA, quality of life); number of medications (e.g. high dose of steroids, chemicals in the body). Table 4 provides random examples in each category.

**Table 4:** Random examples of expressed concerns about side effects of combination medication, divided into 5 categories by 5/18 patients

**Concerns about side effects**	**Patients’ accounts**
Ambivalence	‘I had some concerns about the side effects, but at the same time I had no choice, because that is what I have to take in order to improve my situation… so I was willing to go along with it [medication]’. (Patient 13, early RA)
Physical problems (long-term)	‘I wanted to know whether it would affect the organs in my body, because I have had chest x-rays and things like that, and I have blood test every month. I always worry about what would happen to my inner organs’. (Patient 8, established RA)
Worries about cancer	‘In the back of my mind I am worried about getting a cancer or tumour, because I am thinking I am getting quite immune suppressed.’ (Patient 7, established RA)
Uncertainty about treatment failure	‘I do not find the treatment helped at all, as I still got pain, the drugs are not controlling the RA…I can only see the situation getting worse’. (Patient 15, early RA).
Number of medication	‘I mean at the beginning I was a bit concerned about the medication MTX, ahm… but the ones I am now on, I am OK about.. I just think: there are so many tablets to take’. (Patient 6 early RA)

The majority (11/18) described actual physical and systemic side effects that they experienced while on combination therapy. These related to weight gain and loss, chest infections (in relation to biologics), intestinal problems (e.g. nausea, indigestion), anaemia, fatigue, loss of hair and migraine. Some were of sufficient severity for their medication to be temporarily discontinued. A substantial minority (8/18) needed additional top-up steroid injections, additional blood monitoring and constant adjustment of their medication.

"‘The first thing was visual… it [medication] is changing my appearance so drastically, when I am looking in the mirror I am not happy what I see. I struggle to fit into my clothes that I had for quite some time, it makes me really unhappy, how long is this going to go on?’ (Patient 14, early RA)"

"‘There came a time when I had to stop the medication, because I developed an infection in my chest. So I had to stop the injections and start on antibiotics, but fortunately I started on the injection last week again’. (Patient 4, established RA)"

### Theme 4: Management of combined therapy at home

Most patients received verbal (14/18) and written (17/18) information and communication in forms of leaflets mainly from nurses and also from some doctors in the outpatient clinic prior to their decision to commence on the new combined therapy. All appreciated the dialogue with medical and particularly with nursing staff, as it provided them with sufficient details to reach a joint decision about when and which treatment to start and what kind of practical adjustments were involved.

"‘I got all the information from the nurse, and I read all about the different anti-TNF treatments, and the one with the weekly injections suited me best and it really has helped me’. (Patient 5)"

"‘The staff did go through the [written] information with me; they explained things to me that do really help. It helped me get my head round things, yeah’. (Patient 13)"

1/18 patient reported accessing relevant internet websites for further information. Many (12/18) emphasised and appreciated the support from the clinic staff that provided them with additional relevant and ongoing reassurance. However, 4/18 people criticized how communication with clinic staff was frustrating at times, as appointment times were delayed or misunderstood.

"‘Today I came up to Dr. X’s clinic. I thought that was strange because I am normally under Professor X and I had an injection in my shoulder. But I was not aware that I was having that [injection].. but I was just saying to the receptionist I was waiting for a scan on my shoulder you know… so I was not quite sure what I came up for. (Patient 2)"

All (18/18) patients provided detailed descriptions about how they incorporated and practically managed their combined medication on a daily basis, with some (4/18) including depictions of their individual treatment beliefs. The majority (17/18) managed well in getting into ‘a routine’ with the use of memory aids (6/18) to work around their personal and public engagements, including being taught how to inject medication (biologics) subcutaneously (5/18). For many the issue of medication was either expressed with positive (8/18) or ambivalent attitudes (10/18). About half (7/18) stated that they forgot the name of their past or present medication or found it at times difficult to remember taking the tablets. This seemed particularly true for methotrexate, as the tablets are only taken once a week.

Despite changes in medication, due to unforeseen complications or treatment failures, patients seemed to adapt well. Nevertheless, one patient, recently diagnosed with RA admitted that she found taking her medication and regular blood monitoring ‘a torture’ (see Table 5).

**Table 5:** Random examples of accounts how participants (7/18) manage their combined medication and blood monitoring

**Patient**	**Management of medication (n = 5 positive attitude, n = 10 ambivalent attitude)**
Pilot Patient 1	‘I get used to the tablets and everything and I get used to the injections now as well. So I take MTX on a Wednesday, the injections on a Wednesday and the Folic Acid on a Friday.. I have done it for couple of years now, so I got used to it’.
Patient 3	‘I take one tablet [Leflunomide] every day in the morning and the other one [MTX] I take once a week on Saturdays. When I take MTX, I take it in the morning, and the other one in the afternoon, because I was concerned about mixing both medications at once, so I always keep some time in between. I do not forget the tablets, because once you are sick you know that you depend on this medication, I always remember and my husband is always asking me whether I have taken my medication’.
Patient 5	‘Monday is the day when I take MTX and inject Enbrel under the skin. I call it my ‘bad day’, because I know I have to inject myself, and I hate it. I call it my ‘bad day’ like many working people talk about Monday is their worst day! I take the Folic Acid on Wednesday and that is me done for the rest of the week. The monthly blood taking is the down side of this new treatment, it is a real pain’.
Patient 6	‘I put like a timer on my phone to remind me, so I remember to take them [medication]. I got other tablets to take with it [RA medication], I just find it a lot of medication to be taken every day, as I said I got high blood pressure as well… it is hard, it is kind of like make myself do it…I feel like a walking chemist sometimes’.
Patient 7	‘I know my colleagues laughed when I told them that I went to Boots to buy a Dosette Box. I would be lost without my Dosette Box and ‘Pharmacy to you’, these are my two things…it was easier to have a Dosette Box going on holiday, instead of taking umpteen bottles, and keeping it away from a toddler; when I go out for dinner, I grab my evening tablets all at once with me in the handbag’.
Patient 14	‘It is a torture really, because I struggle a lot to get the medications out [of the package], even though I get help sometimes’.
Patient 16	‘The blood monitoring is easy; I just go to the GP. For my injections I actually went to the hospital and was taught how to do it and so that is all fine, no problem’.

One patient (Pilot Patient 2) described how he had kept some injections (biologic) at home, which he was advised by the doctor to discontinue due to a chest infection. At times when he felt unwell, once every two months, he self-medicated, because as he reported ‘I knew the result would be good, you know’.

## Discussion

To our knowledge this is the first qualitative study that examined systematically in patients with early and established RA their expectations, impact on quality of life, concerns about side effects and the management of the treatment when taking combined medication with DMARDs or DMARDs and biologics. There was no notable difference by gender, ethnicity, age and disability in patients’ accounts.

Decisions by medical staff to prescribe combined therapy, according to the patients’ understanding, were ‘the disease was not well controlled’ and ‘monotherapy was not effective enough’ were patients’ views and show the diversity of expressions used. Although the notions are related they may not mirror the medical categories used, e.g. no response, incomplete response or response to treatment.

Within our analytical framework three key messages emerged that appear relevant for clinical practice. Firstly, patients’ expectations about the combined treatment focused mainly on their physical symptoms and concerns about the side effects. Secondly, patients reported that the efficacy of the treatment and its subsequent benefit on their quality of life was mixed. A subgroup of patients on DMARD and biologic seemed to be particularly satisfied with the treatment offered and their quality of life. Finally most patients were able to manage their combined treatment at home, for some with additional electronic or memory aids in close ongoing dialogue and support from the clinic staff. This was also highlighted in the literature [28].

During the interviews many patients alluded to the diverse impact that RA has on their lives with its fluctuating nature. However, we decided to exclude these accounts from this paper, as published elsewhere [28,29] and to report the key messages that emerged from the interviews.

The introduction of DMARDs when used in combination are prescribed in varying speed during consultations, depending on patients’ medical and emotional circumstances, with the need to continually modify DMARD treatment. These may result in a range of disease activity scores and influence patients’ perceptions. Moreover, intermittent gluco-corticoide treatment is often used as part of intensive combination treatment that may influence patients’ views.

One published qualitative interview study focusing on patients’ perception following commencement of anti-TNF therapy [8] reached similar findings, e.g. how physical functions may improve and high expectations of some patients were not always met. Given the long-term disabling effect of RA, the guidelines of National Institute for Health and Clinical Excellence (NICE) [30] recommended the benefits of particular combined DMARDs in early and established RA. In addition “treat to target” is now commonly recommended and its use is supported by a number of studies [31].

Our findings resonate broadly with the collaborative management of chronic illness model [23] that consists of four elements, e.g. (i) collaborative definition of problems, (ii) goal setting and planning; (iii) training and support in self-management and (iv) active and sustained follow-up. Patients’ contribution to their self-care and its medical care are clearly complementary, rather than in competition. Both parties depend on each other for reaching the goal of controlling the disease activity, joint erosion and pain, including the management of their daily activities of living and medication, within the context of patients’ lives.

What von Korff’s elements’ of care framework offer is an improvement of long-term illness in a structured and explicit way. Within the context of our study findings, three of the four aspects of the model were adhered to by staff and patients, whereas overt goal setting and planning did not receive much attention. However it has become evident from von Korff’s publications in chronic disease management that an effective collaborative care approach strengthens and supports patients’ self-care in long-term disease such as Rheumatoid Arthritis, including their family members and carers.

### Strengths and limitations of the study

The qualitative methodology adopted for the data gathering has been advantageous, as patients provided first hand and detailed views what matters most to them and what they have difficulties with in relation to their combination therapy.

One limitation was that the study cohort was recruited from one large inner city outpatient clinic; where patients attend from a wide geographical area and socio-demographically diverse background, due to its tertiary status. Moreover, the findings may not be fully generalisable without replication, but they are timely and extend further knowledge of how patients’ cope and manage with an ever changing treatment approach in rheumatology care.

Finally, for reasons of lack of resources for language interpretation, we included in the study patients who could be interviewed in English, even if there use of English was limited. We estimate that fewer than 5% of all patients attending this Rheumatology clinic cannot speak English sufficiently to communicate with staff, so that in our view this inclusion criterion did not lead to substantial selection bias in the study and did lead to a representative sample of all clinic patients.

## Conclusions

The treatment of patients with RA is in a stage of constant flux, due to new emerging evidence about treatment modalities. At each stage of such treatment alterations, obtaining patients’ agreement will be imperative, as they also need to understand and ideally adapt to these changes. As is well known within clinical practice, medication concordance is a complex and highly personal issue that needs careful consideration when prescribing medication in close collaboration and concurrence with patients.

## Appendix 1

### Interview Guide

In this interview I would like to explore three key areas: why you started on the new treatment, what is your understanding of the treatment and is your quality of life different since you started treatment? At the end I would welcome any additional information which I may have left out but which are relevant to your experiences, including your thoughts about taking part in a study such as this. The interview may take up to 30 minutes and can be paused at any time. If you provide information you do not wish to have recorded, the tape recorder can be stopped at any time and restarted with your agreement.

1. What led to the decision to start you on combination therapy?

● Can you describe your feelings about starting the combined treatment?

2. What is your understanding of the combined treatment?

● What type of information did you receive about the combination therapy and by whom?

● What expectations do you have from the combination treatment? What outcomes are important?

● What makes you satisfied and dissatisfied with the treatment? e.g. range of side effects, how much do you disclose side effects of the drugs to clinic staff and family.

● How do you manage with the prescribed regimes, e.g. administration, blood monitoring.

● What are your attitudes/concerns towards the medication? In which way to they differ from the time before you started combination therapy?

● How do you decide that the treatment is working?

3. How do you assess your quality of life since you commenced the combination therapy?

● Any difference from the time before when receiving one medication for your RA?

4. Any other issues you would like to add that were not covered with the questions above?

## Competing interests

The authors declare that they have no competing interests.

## Authors’ contributions

HL: had main responsibility for conducting the interviews and transcribing, the data analysis, to write the first draft of the paper and for overseeing the submission process. DH: contributed to the transcribing of the interviews and editing of the paper. SLH: contributed to the data analysis and editing of the paper. DLS: proposed the idea of the study and contributed to the editing of the paper. All authors have seen and approved the final version of the paper before submission.

## Pre-publication history

The pre-publication history for this paper can be accessed here:

http://www.biomedcentral.com/1471-2474/13/200/prepub
